# Time to Surgery Does Not Affect Overall or Disease-Free Survival of Patients with Primary Resectable PDAC

**DOI:** 10.3390/jcm11154433

**Published:** 2022-07-29

**Authors:** Anne Jacobsen, Mirianna Hobbs, Susanne Merkel, Anke Mittelstädt, Franziska Czubayko, Christian Krautz, Georg F. Weber, Robert Grützmann, Maximilian Brunner

**Affiliations:** Department of General and Visceral Surgery, Friedrich-Alexander-University (FAU Erlangen-Nuremberg), Krankenhausstrasse 12, 91054 Erlangen, Germany; anne.jacobsen@uk-erlangen.de (A.J.); mirianna.hobbs@extern.uk-erlangen.de (M.H.); susanne.merkel@uk-erlangen.de (S.M.); anke.mittelstaedt@uk-erlangen.de (A.M.); franziska.czubayko@uk-erlangen.de (F.C.); christian.krautz@uk-erlangen.de (C.K.); georg.weber@uk-erlangen.de (G.F.W.); robert.gruetzmann@uk-erlangen.de (R.G.)

**Keywords:** pancreatic ductal adenocarcinoma, primary resection, overall survival, disease-free survival, time to surgery

## Abstract

(1) Background: Delay in therapy for pancreatic ductal adenocarcinoma (PDAC) may contribute to a worse outcome. The aim of this study was to investigate the prognostic value of time from diagnosis to surgery in patients undergoing upfront surgery for primarily resectable pancreatic carcinoma. (2) Methods: This retrospective single-center study included 214 patients who underwent primary resection of PDAC from January 2000 to December 2018 at University Hospital Erlangen. Using a minimum *p*-value approach, patients were stratified according to time to surgery (TtS) into two groups: TtS ≤ 23 days and TtS > 23 days. Postoperative outcome and long-term survival were compared. (3) Results: Median TtS was 25 days. The best cut-off for TtS was determined as 23 days. There were no differences regarding postoperative outcome or overall survival (OS) and disease-free survival (DFS) (OS: 23.8 vs. 20.4 months, *p* = 0.210, respectively, and DFS: 15.8 vs. 13.6 months, *p* = 0.187). Multivariate analysis revealed age, lymph node metastasis, tumor differentiation and resection status as significant independent prognostic predictors for OS and DFS. (4) Conclusions: A delay of surgery > 23 days after first diagnosis does not affect overall or disease-free survival of patients with primary resectable PDAC. However, the psychological impact of a delay to patients waiting for surgery should not be underestimated.

## 1. Introduction

Pancreatic ductal adenocarcinoma (PDAC) is a leading cause of cancer-related mortality worldwide, with a mortality rate almost as high as its incidence [1,2]. The poor prognosis is reflected in a 5-year overall survival of about 10% [3]. Nowadays, the therapy for pancreatic adenocarcinoma always includes multimodal therapy concepts. Nevertheless, surgical radical resection remains the decisive component of a potentially curative therapy. In patients with resectable pancreatic adenocarcinoma, upfront surgery is still the therapy of choice, even if current studies examine and propagate the value of neoadjuvant therapy in primarily resectable pancreatic carcinoma [4]. However, more than 70% of patients diagnosed with pancreatic cancer are not eligible for resection due to metastases or locally advanced cancer [5].

The high rate and early occurrence of metastases, the rapid progression, as well as the associated high mortality rate of pancreatic carcinoma suggest that therapy for pancreatic carcinoma may be time-critical [6]. Delays between the diagnosis of pancreatic carcinoma and the start of appropriate therapy can occur for a variety of reasons, such as the need for a biliary stenting or patient-related factors, such as processing the diagnosis or fear of further therapy, but these delays can have a negative impact on the outcome. A prolonged time to surgery in patients with resectable pancreatic adenocarcinoma could hypothetically lead to tumor progression and, therefore, worse prognosis.

Recent investigations on the impact of time to surgery on the prognosis of patients with resectable pancreatic adenocarcinoma are inconsistent. Most studies found no impact of a time delay between diagnosis and surgery on survival [7,8,9,10]. However, there is some evidence that patients with PDAC < 20 mm benefit from surgery within 30 days after diagnosis [11]. Moreover, Sanjeevi et al. found that an interval > 32 days between diagnosis and surgery leads to a higher risk of progression to an unresectable tumor stage [12].

The aim of this study was to evaluate the impact of the time interval between first diagnostic imaging with suspected pancreatic carcinoma and surgery on overall survival and disease-free survival in patients with primarily resectable PDAC.

## 2. Materials and Methods

The prospectively maintained Erlangen Cancer Registry of the Department of Surgery was used to identify patients for this retrospective analysis. All adult patients with primary resectable PDAC who underwent upfront surgery at University Hospital Erlangen between 1 January 2000 and 31 December 2018 were included in this study. All patient cases were discussed in an interdisciplinary tumor board, and the pancreatic malignancy was classified as primary resectable based on the available diagnostics. Patients with tumors classified as borderline resectable according to our interdisciplinary tumor board received neoadjuvant therapy. Exclusion criteria comprehend the performance of any neoadjuvant therapy and missing data regarding the first imaging and diagnosis.

Patients’ clinical data, including the date of first diagnosis, were retrieved from the clinical information system. First diagnosis was defined as the date of the first imaging with urgent suspicion of a malignant pancreatic tumor. Time to surgery (TtS) was defined as the time between first diagnosis and the date of surgery. Patients’ pathological and survival data were obtained from the Erlangen Cancer Registry of the Department of Surgery. The TNM Classification of malignant tumors as presented by the Union for International Cancer Control (UICC) (according to the 8th edition from 2017) was used to describe the histopathological details [13]. Morbidity was evaluated by Clavien–Dindo classification [14]. Major morbidity was defined as Clavien–Dindo III, IV and V. Postoperative pancreatic fistula (POPF), delayed gastric emptying (DGE) and post-pancreatectomy hemorrhage (PPH) were defined according to the definitions of the International Study Group of Pancreatic Surgery (ISGPS) [15,16,17]. The median follow-up time for the patients was 19.0 months (range 0–194 months).

Using this dataset, a threshold analysis using a minimum *p*-value approach was performed to determine the best cut-off for time to surgery (TtS) regarding overall survival (Supplementary Table S1). Using the identified cut-off, patients with shorter TtS were compared with the group of patients with longer TtS.

This retrospective study was approved by the local ethics committee (22-165-Br).

### 2.1. Surgical Procedures and Postoperative Course

All surgical procedures were performed by experienced visceral surgeons with many years of practical experience in pancreatic surgery. All procedures included an adequate oncological lymphadenectomy. Depending on the tumor localization, different surgical procedures were performed. Pancreatic head resections always included an interaortocaval lymph node dissection. Interaortocaval lymph nodes do not belong to the locoregional lymph nodes of the pancreas and are therefore to be evaluated as M1 in the case of tumor involvement. Therefore, there are some patients classified as pM1 who received primary resection. In the case of intraoperative evidence of liver metastases or peritoneal carcinosis, no resection of the primary tumor was performed. Additional venous vascular resections, as well as multi-visceral resections, were performed if necessary for archiving R0 situation. Arterial vascular resection was only carried out in exceptional cases.

Adjuvant chemotherapy was generally recommended to all patients except those with a significantly reduced postoperative general condition. Some patients refused an adjuvant chemotherapy. Depending on the condition of the patient, adjuvant chemotherapy was performed with either gemcitabine or 5-FU-based chemotherapy. Regular follow-up including computer tomography (CT) of the thorax and abdomen was recommended to all patients starting quarterly and then every six months from the 3rd year.

### 2.2. Statistical Analysis

SPSS^®^ Version 28 (IBM, Armonk, NY, USA) was used to analyze the data. Comparisons of metric and ordinal data were calculated with the Student’s t-test or Mann–Whitney U test. The Chi-squared test was used for categorical data. The minimum p-value approach was used to determine the optimal cut-off for time to surgery (TtS). Overall survival (OS) and disease-free survival (DFS) were respectively calculated for the period between the date of surgery and the date of death, date of local or distant recurrence or date of last follow-up. For survival analysis, seven patients were excluded because of perioperative death. Possible factors related to the overall (OS) and disease-free survival (DFS) of patients were tested using univariate and multivariate analysis. Variables with a *p* ≤ 0.1 in univariate analysis were used for multivariate analysis using a Cox regression model. Survival curves were plotted using the Kaplan–Meier method and compared with the log-rank test. A *p*-value ≤ 0.05 was considered statistically significant.

## 3. Results

### 3.1. Dataset of Patients

A total of 214 patients underwent primary pancreatic resection because of pancreatic ductal adenocarcinoma between 1 January 2000 and 31 December 2018 at the University Hospital Erlangen. Of these, 22 patients had to be excluded because of missing data for the first imaging. Thus, 192 patients remained for this analysis.

### 3.2. Time to Surgery

**Figure 1:**
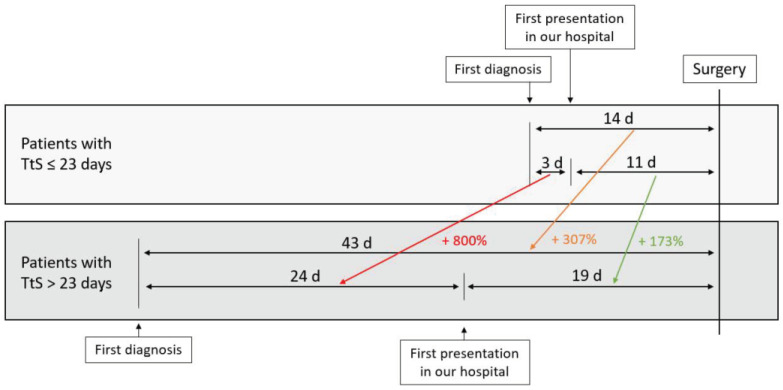
Mean time (in days (d)) between first imaging and surgery, and first admission in our hospital and surgery, stratified for patients with time to surgery (TtS) ≤ 23 days vs. > 23 days.

The mean and median times between first diagnosis and surgery were 29 days (+/− 20 days) and 25 days (range 1–119 days), respectively. The distribution of patients according to time to surgery is shown in Table 1. The minimum *p*-value approach revealed that 23 days between first imaging and surgery was the optimum threshold to analyze the impact of a shorter (group 1) or a longer (group 2) time interval on overall survival (Supplementary Table S1). The mean time to surgery was 14 days in group 1 compared to 43 days in group 2. Analysis of delay reasons especially revealed an eight-fold time interval between first diagnosis and first presentation in our hospital in group 2 (TtS > 23 days) compared to group 1 (Figure 1).

### 3.3. Patient Characteristics

Of the 192 included patients (median age: 68 years, 49% female), 90 patients had a TtS ≤ 23 days (group 1), whereas 102 patients underwent surgery 24 days or later after first diagnosis (group 2). Patient characteristics including ASA score, comorbidities, preoperative biliary stenting and preoperative laboratory did not differ between the two groups (Table 2).

### 3.4. Surgical and Histopathological Details

Pancreatic head resection was performed most frequently (75%), followed by distal pancreatectomy (21%) and total pancreatectomy (4%). Additional vascular and multi-visceral resection was required in 30% and 18% of patients, respectively. R0 resection was achieved in 88% of the patients.

The surgical and histopathological details, including the TNM stage and the R-status of the patients, were similar among the two groups (Table 3).

### 3.5. Short-Term Postoperative Outcome Parameters

The postoperative outcome parameters are shown in Table 4. Regarding in-hospital morbidity, including POPF, DGE and PPH as well as re-operation rate and in-hospital mortality, there was no significant difference between the two groups. Both groups had a median length of postoperative stay of 18 days (*p* = 0.366). In total, 55% of the patients received an adjuvant chemotherapy, with a slightly higher rate in group 2 (57% vs. 52%, *p* = 0.534) (Table 4).

### 3.6. Overall and Disease-Free Survival

**Figure 2:**
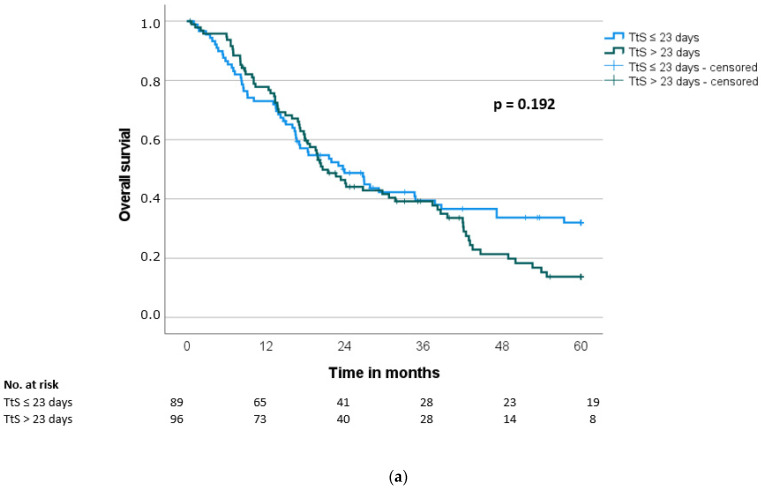

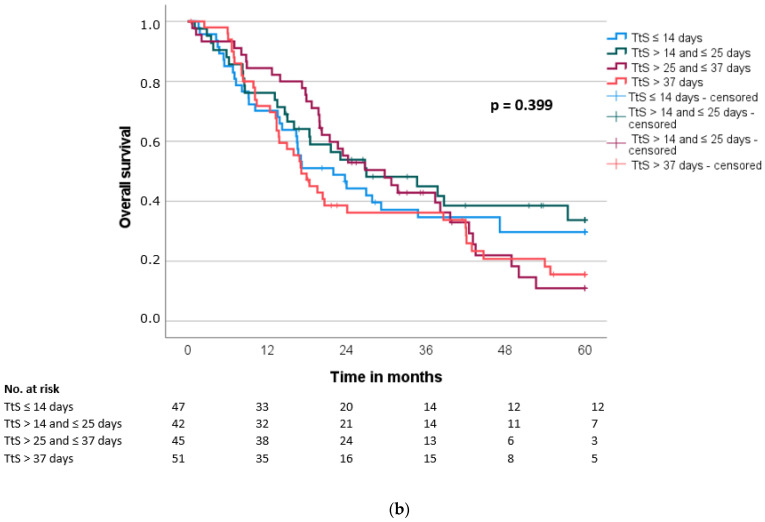
(**a**) Overall survival (OS) according to time to surgery (TtS) (≤23 days vs. >23 days). (**b**) Overall survival (OS) according to time to surgery (TtS) (≤14 days (first 25% quartile) vs. >14 and ≤25 days (second 25% quartile) vs. >25 and ≤37 days (third 25% quartile) vs. >37 days (fourth 25% quartile)). Seven patients were not included in the survival analysis because of perioperative death.

**Figure 3:**
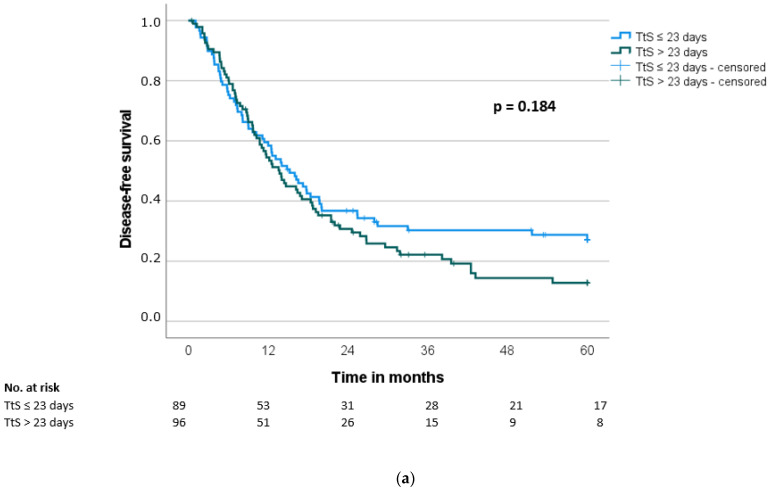

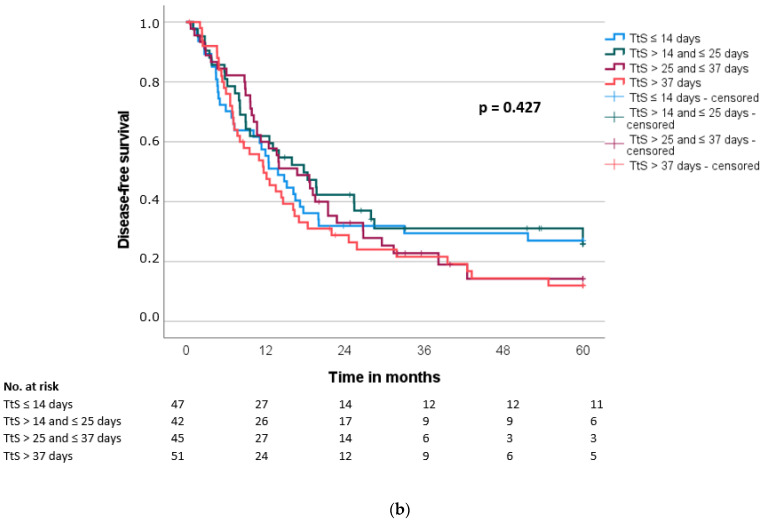
(**a**). Disease-free survival (DFS) according to time to surgery (TtS) (≤23 days vs. >23 days). (**b**) Disease-free survival (DFS) according to time to surgery (TtS) (≤14 days (first 25% quartile) vs. >14 and ≤25 days (second 25% quartile) vs. >25 and ≤37 days (third 25% quartile) vs. >37 days (fourth 25% quartile)). Seven patients were not included in the survival analysis because of perioperative death.

The median overall survival (OS) and disease-free survival (DFS) were 21.5 ± 2.0 months and 13.7 ± 1.5 months, respectively. Both the overall survival and the disease-free survival showed no significant difference between the groups (OS: 24.0 months vs. 20.7 months, *p* = 0.192; DFS: 15.2 months vs. 13.6 months, *p* = 0.187) (Table 4, Figure 2a and Figure 3a). Using stratification of patients in quartiles of time to surgery, there was again no difference between the groups (OS: *p* = 0.399; DFS: *p* = 0.427) (Figure 2b and Figure 3b).

### 3.7. Prognostic Factors for Overall and Disease-Free Survival

Potentially prognostic factors of patients with resected pancreatic carcinoma regarding OS and DFS are presented in Table 5. Multivariate analysis revealed that age (OS: hazard ratio (HR) 2.39, *p* < 0.001; DFS: HR 1.51, *p* = 0.032), lymph node metastasis (OS: HR 2.00, *p* < 0.001; DFS: HR 1.78, *p* = 0.005), R-status 1 or 2 (OS: HR 2.69, *p* = 0.001; DFS: HR 1.74, *p* = 0.050) and differentiation with a grading of 3 (OS: HR 1.84, *p* = 0.003; DFS: HR 1.62, *p* = 0.017) were significant independent prognostic factors regarding OS as well as DFS (Table 5).

## 4. Discussion

A delay in treatment of pancreatic ductal adenocarcinoma may contribute to poor prognosis. In our single-center retrospective study including 192 patients who underwent primary surgery for pancreatic ductal adenocarcinoma, the time interval between first diagnostic imaging and surgery did not affect overall survival and disease-free survival.

The influence of time delays between diagnosis and treatment of pancreatic cancer was the topic of research in a few studies. The results of the present study are consistent with those of most others, which found no impact of a time delay to surgery on overall survival [7,8,9,10,11,18,19,20]. However, there are some studies that have shown higher resectability rates in patients with pancreatic cancer when the time delay to surgery is reduced. Glant et al. described a higher risk of unknown metastases encountered during surgery in patients with proximally located PDAC and longer intervals between the last diagnostic imaging and surgery [21]. Roberts et al. showed that avoiding preoperative biliary drainage in PDAC patients leads to shorter time intervals between diagnostic imaging and surgery and to more potentially curative resections [22]. In addition, Sanjeevi et al. reported a doubled risk for an unresectable tumor when the time to surgery exceeded 32 days [12]. However, none of these studies analyzed survival rates. Thus, it remains unclear whether the findings of these mentioned studies were also reflected in a better oncological outcome.

However, there are several aspects to consider concerning the time to surgery: First, in addition to the time interval for surgery itself, the reason for the delay may also play a decisive role. One of the most important reasons for delaying surgery is performing preoperative biliary drainage, although some recent studies have shown that this can increase the postoperative complication rate and that early surgery is safe in the presence of jaundice up to a bilirubin of 250 µmol/L [23,24,25]. However, the rate of patients with preoperative biliary stenting did not differ in our cohort. In the group of delayed surgery, our analysis showed, in particular, a delay in the time interval between initial diagnosis and presentation in our hospital, which could have different explanations: Either uncertainty in diagnostics led to further investigations that required more time, or there was a delay of patient referral to the pancreas center, or patient-related aspects such as processing of the diagnosis or respect for further therapy led to a delayed admission. On the other hand, there may also be patients who can benefit from the delay to surgery: Patients with a poor general condition at the time of diagnosis could be brought to an operable status by intensified prehabilitation, including nutritional therapy. Moreover, a delay caused by additional diagnostic measurements such as liver-MRI can lead to a better selection of patients who may benefit from surgical exploration.

Second, there is likely to be a selection bias when determining the time to surgery since, for example, patients with a larger tumor appear more urgent and are therefore operated on earlier but have a poorer prognosis due to the advanced tumor. However, TNM classification did not differ between groups.

Third, the question is raised whether patients who develop metastasis or a progression to local irresectability within some days would really benefit from resection. Using a longer time interval between diagnosis and resection may give the chance to select these patients before undergoing surgery, as even R0 resection would not improve their survival but carries the risk of possible postoperative complications, which in turn delays the necessary chemotherapy.

Fourth, there is a relevant psychological impact of delayed surgery on cancer patients that may lead to a seriously impaired quality of life by waiting for surgery [26].

Fifth, patients with delayed surgery need to be re-differentiated based on the exact time interval of delay, since a delay in the range of 25–50 days could have a different impact compared to a delay of 90–120 days. However, due to the limited number of patients in the group with the longest delays to surgery, a possible effect cannot be meaningfully investigated in our collective. Using stratification in quartiles (Figure 2b and Figure 3b), there is no significant difference in survival for the quartile with a time to surgery > 37 days compared to the other quartiles.

Moreover, upfront surgery for primarily resectable pancreatic carcinoma is currently under scrutiny, as recent studies show improved survival through neoadjuvant chemotherapy even in the case of primarily resectable tumors [4]. Our results cannot be extrapolated to patients with neoadjuvant therapy. Therefore, if neoadjuvant therapy becomes the standard of care for patients with primarily resectable pancreatic carcinoma in the future, the current data will lose their value.

The present study has several limitations. First, it is a single-center study that has the advantage of a homogeneous therapy concept but makes it difficult to generalize the results. Second, the retrospective analysis of prospective recorded data may have incurred some bias. Third, the number of patients is limited and was collected over a long period of 18 years. Therapy of pancreatic carcinoma changed over the years, especially concerning adjuvant chemotherapy. However, there was no significant difference between the groups regarding the year of surgery.

## 5. Conclusions

The present study confirms current data that the time to surgery does not affect overall or disease-free survival of patients with primary resectable PDAC. However, the psychological impact of delayed surgery should also be considered, as the quality of life of cancer patients is seriously impaired by waiting for surgery.

## Figures and Tables

**Table 1:** Distribution of patients undergoing pancreatic resection for pancreatic ductal adenocarcinoma according to time to surgery (TtS) (days) (*n* = 192).

	Number (*n*)	% of All Patients
**TtS ≤ 10 days**	26	13.5
**TtS > 10 and ≤ 20 days**	51	26.6
**TtS > 20 and ≤ 30 days**	49	25.5
**TtS > 30 and ≤ 40 days**	29	15.1
**TtS > 40 and ≤ 50 days**	17	8.9
**TtS > 50 and ≤ 60 days**	4	2.1
**TtS > 60 and ≤ 90 days**	11	5.7
**TtS > 90 and ≤ 120 days**	5	2.6

**Table 2:** Characteristics of patients undergoing pancreatic resection for pancreatic ductal adenocarcinoma stratified to time to surgery (TtS) (≤23 days vs. >23 days).

	TtS ≤ 23 Days	TtS > 23 Days	*p*-Value
**Number**	90	102	
**Age (years), median (IQR)**	68 (15)	69 (14)	0.433
**Gender, *n* (%)**			1.000
**Female**	44 (49)	50 (49)	
**Male**	46 (51)	52 (51)	
ASA (n = 180) *, *n* (%)			0.769
**I**	2 (2)	1 (1)	
**II**	51 (61)	61 (64)	
**III**	31 (37)	34 (35)	
**BMI (kg/m^2^), median (IQR)**	25.5 (4.4)	25.6 (6.4)	0.965
Alcohol abuse (*n* = 169) *, *n* (%)	44 (56)	43 (47)	0.280
Nicotine abuse (*n* = 189) *, *n* (%)	19 (22)	23 (23)	0.863
**Comorbidity, *n* (%)**			
**Hypertension**	48 (53)	62 (61)	0.310
**Diabetes**	20 (22)	34 (33)	0.108
**Cardiovascular**	10 (11)	14 (14)	0.665
**Pulmonary**	8 (9)	9 (9)	1.000
**Cerebrovascular**	4 (4)	7 (7)	0.546
**Liver disease**	7 (8)	9 (9)	1.000
**Preoperative biliary stenting, *n* (%)**	45 (50)	56 (55)	0.561
**Preoperative hemoglobin (g/dL), median (IQR)**	12.9 (2.4)	12.7 (2.3)	0.587
**Preoperative WBC (10^9^/L), median (IQR)**	6.8 (3.2)	7.2 (3.6)	0.402
**Preoperative albumin (g/L), median (IQR)**	40.4 (6.6)	40.1 (7.8)	0.954
**Preoperative CRP (mg/L), median (IQR)**	8 (20)	5 (15)	0.140
Preoperative CA19-9 (U/mL) (*n* = 174) *, median (IQR)	118 (619)	88 (213)	0.265
Preoperative CEA (ng/mL) (*n* = 137) *, median (IQR)	3.0 (3.9)	2.3 (3.3)	0.197

IQR = interquartile range; ASA = American Society of Anesthesiologists classification; BMI = body mass index; WBC = white blood cells; CRP = C-reactive protein. * Missing data.

**Table 3:** Surgical and histopathological details of patients undergoing pancreatic resection for pancreatic ductal adenocarcinoma stratified to time to surgery (TtS) (≤23 days vs. >23 days).

	TtS ≤ 23 Days (*n* = 90)	TtS > 23 Days (*n* = 102)	*p*-Value
**Kind of surgery**			0.743
**Pancreatic head resection**	69 (77)	76 (74)	
**Distal pancreatectomy**	17 (19)	23 (23)	
**Total pancreatectomy**	4 (4)	3 (3)	
**Portal vein resection, *n* (%)**	26 (29)	30 (29)	1.000
**Arterial resection, *n* (%)**	3 (3)	1 (1)	0.342
**Multi-visceral resection, *n* (%)**	19 (21)	15 (15)	0.262
**Operative time (min), median (IQR)**	280 (101)	278 (108)	0.494
**Intraoperative blood loss (mL), median (IQR)**	600 (575)	500 (700)	0.809
**Intraoperative blood transfusion, *n* (%)**	23 (26)	31 (30)	0.521
**T category**			0.664
**pT1**	4 (4)	8 (8)	
**pT2**	16 (18)	20 (20)	
**pT3**	68 (76)	73 (72)	
**pT4**	2 (2)	1 (1)	
**n category**			0.662
**pN0**	35 (39)	43 (42)	
**pN+**	55 (61)	59 (58)	
**M category**			0.621
**pM0**	83 (92)	91 (89)	
**pM1**	7 (8)	11 (11)	
**R-status**			0.687
**R0**	78 (87)	90 (88)	
**R1**	8 (9)	10 (10)	
**R2**	4 (4)	2 (2)	
**Differentiation**			0.550
**G1**	3 (3)	1 (1)	
**G2**	30 (33)	35 (34)	
**G3**	57 (63)	66 (65)	

**Table 4:** Outcome parameter of patients undergoing pancreatic resection for pancreatic ductal adenocarcinoma stratified to time to surgery (TtS) (≤23 days vs. >23 days).

	TtS ≤ 23 Days (*n* = 90)	TtS > 23 Days (*n* = 102)	*p*-Value
**Morbidity, *n* (%)**	56 (62)	62 (61)	0.882
**Major morbidity, *n* (%)**	22 (24)	32 (31)	0.336
**Mortality, *n* (%)**	1 (1)	6 (6)	0.123
**Re-operation, *n* (%)**	8 (9)	10 (10)	1.000
**POPF, *n* (%)**	13 (14)	21 (21)	0.344
**DGE, *n* (%)**	32 (36)	29 (28)	0.352
**PPH, *n* (%)**	1 (1)	0 (0)	0.469
**Surgical site infection, *n* (%)**	8 (9)	4 (4)	0.232
**Length of postoperative stay (days), median (IQR)**	18 (15)	18 (12)	0.366
**Adjuvant chemotherapy, *n* (%)**	47 (52)	58 (57)	0.534
Overall survival (months) *, median (SD)	24.0 (4.2)	20.7 (2.1)	0.192
Disease-free survival (months) *, median (SD)	15.2 (2.4)	13.6 (1.5)	0.187

POPF = postoperative pancreatic fistula; DGE = delayed gastric emptying; PPH = post-pancreatectomy hemorrhage; IQR = interquartile range; SD = standard deviation. * Seven patients were not included in the survival analysis because of perioperative death.

**Table 5:** Prognostic factors of patients with resected pancreatic ductal adenocarcinoma for overall survival (OS) and disease-free survival (DFS).

		Overall Survival (OS) *					Disease-Free Survival (DFS) *				
		Univariate		Multivariate			Univariate		Multivariate		
	*n*	Median OS	*p*	HR	95% CI	*p*-Value	Median DFS	*p*	HR	95% CI	*p*-Value
**Age**			0.002	2.39	1.63–3.49	<0.001		0.054	1.51	1.04–2.19	0.032
**≤70 years**	108	37.4					16.5				
**>70 years**	77	17.9					11.0				
**Gender**			0.505					0.764			
**Female**	92	26.8					13.7				
**Male**	93	21.5					14.4				
ASA (*n* = 174) **			0.035	1.27	0.87–1.86	0.210		0.053	1.16	0.79–1.70	0.444
**I/II**	112	26.8					15.2				
**III**	62	16.9					11.1				
Ca19-9 (*n* = 170) **			0.197					0.070	1.01	0.69–1.50	0.945
**<50 U/mL**	64	24.2					16.8				
**≥50 U/mL**	106	22.7					12.5				
**Interval to surgery**			0.192					0.184			
**≤23 days**	89	24.0					15.2				
**>23 days**	96	20.7					13.7				
**Kind of surgery**			0.333					0.395			
**Pancreatic head resection**	141	24.1					16.2				
**Distal pancreatectomy**	38	18.0					10.7				
**Total pancreatectomy**	6	6.7					6.7				
**Vascular resection**			0.190					0.260			
**Yes**	54	17.2					9.7				
**No**	131	23.7					15.2				
**Multi-visceral resection**			0.329					0.316			
**Yes**	30	18.0					9.7				
**No**	155	24.0					14.7				
**T category**			0.011	1.36	0.83–2.15	0.234		0.004	1.32	0.83–2.09	0.243
**pT1/pT2**	47	37.8					22.8				
**pT3/pT4**	138	19.9					12.5				
**N category**			<0.001	2.00	1.34–3.00	<0.001		<0.001	1.78	1.19–2.67	0.005
**pN0**	77	39.7					19.2				
**pN+**	108	18.4					12.5				
**M category**			0.032	0.74	0.37–1.50	0.405		0.158			
**M0**	168	24.0					14.8				
**pM1**	17	12.7					9.6				
**R-status**			<0.001	2.69	1.48–4.89	0.001		0.013	1.74	1.00–3.02	0.050
**R0**	162	24.2					16.0				
**R1/R2**	23	10.2					8.8				
**Differentiation**			<0.001	1.84	1.23–2.76	0.003		< 0.001	1.62	1.09–2.40	0.017
**G1/G2**	68	38.2					20.0				
**G3**	117	17.2					12.2				
**Morbidity**			0.323					0.737			
**Yes**	111	20.4					14.0				
**No**	74	31.7					14.0				
**Re-operation**			0.334					0.683			
**Yes**	16	17.2					14.0				
**No**	167	23.4					14.0				
**Adjuvant chemotherapy**			0.721					0.981			
**Yes**	105	23.8					14.8				
**No**	80	18.5					12.2				

ASA = American Society of Anesthesiologists classification. * Seven patients were not included in the survival analysis because of perioperative death. ** Missing data.

## Data Availability

All data generated or analyzed during this study are included in this published article. The publication contains one supplemental table.

## Supplementary Materials

The following supporting information can be downloaded at: https://www.mdpi.com/article/10.3390/jcm11154433/s1. Table S1: Minimum *p*-value approach for influence of the time interval between first imaging, where a suspicious pancreatic lesion was observed, to surgery on overall survival (OS) (*n* = 192).
