# Supplementary material for: Time to Surgery Does Not Affect Overall or Disease-Free Survival of Patients with Primary Resectable PDAC
Source: J Clin Med. 2022 Jul 29;11(15):4433. doi: 10.3390/jcm11154433 (PMC9369379; doi:10.3390/jcm11154433)
Supplement: Supplementary file 1 [file jcm-11-04433-s001.zip › jcm-1849604-supplementary.pdf]

Supplement Table. S1: Minimum p-value approach for influence of the time interval between first imaging, where a suspicious pancreatic lesion was observed, to surgery on overall survival (OS) (n = 192).

		Lower time		Higher time	
**Evaluated cut-off (days)**	***p*-value**	**n**	**Overall survival (OS) (months), median (SD)**	**n**	**Overall survival (OS) (months), median (SD)**
≤ 14 (25%-quartile)	0.657	48	17.2 (4.1)	144	21.5 (2.8)
≤ 15	0.454	54	18.5 (4.2)	138	21.5 (2.6)
≤ 20	0.434	77	21.6 (3.8)	115	21.5 (2.8)
≤ 21	0.261	80	21.6 (3.5)	112	20.7 (2.1)
≤ 22	0.124	87	23.8 (4.7)	105	20.3 (2.2)
**≤ 23**	**0.094**	**90**	**23.8 (4.2)**	**102**	**20.3 (2.1)**
≤ 24	0.182	94	23.4 (4.3)	98	20.3 (2.0)
≤ 25 (median)	0.199	97	23.4 (4.3)	95	20.3 (2.0)
≤ 26	0.177	100	23.8 (3.9)	92	20.3 (2.0)
≤ 27	0.317	107	23.1 (3.7)	85	20.4 (2.8)
≤ 28	0.269	113	23.1 (3.5)	79	20.4 (2.9)
≤ 29	0.481	117	22.0 (2.5)	75	20.7 (3.1)
≤ 30	0.097	126	23.8 (3.2)	66	19.6 (1.7)
≤ 31	0.125	131	23.8 (3.3)	61	18.0 (1.8)
≤ 32	0.105	135	24.0 (3.3)	57	17.3 (1.9)
≤ 33	0.138	136	24.0 (3.1)	56	18.0 (1.6)
≤ 34	0.177	138	23.8 (2.8)	54	18.0 (2.1)
≤ 35	0.194	140	23.8 (2.8)	52	17.2 (2.0)

≤ 37 (75%-quartile)	0.260	149	23.1 (2.7)	43	17.1 (2.1)
≤ 40	0.787	155	21.6 (2.9)	37	19.6 (4.0)

SD = Standard Deviation.
